# Prevalence, incidence estimations, and risk factors of *Toxoplasma gondii* infection in Germany: a representative, cross-sectional, serological study

**DOI:** 10.1038/srep22551

**Published:** 2016-03-03

**Authors:** Hendrik Wilking, Michael Thamm, Klaus Stark, Toni Aebischer, Frank Seeber

**Affiliations:** 1 Unit for Gastrointestinal Infections, Zoonoses and Tropical Infections, Robert Koch Institute, 13353 Berlin, Germany; 2 Central Epidemiological Laboratory, Robert Koch Institute, 13302 Berlin, Germany; 3 Unit for Mycotic and Parasitic Agents and Mycobacteria, Robert Koch Institute, 13353 Berlin, Germany

## Abstract

Representative data on the extent of endemicity, burden, and risk of human toxoplasmosis are scarce. We assessed the prevalence and determinants of seropositivity of *Toxoplasma gondii* among adult participants of a nationwide representative cross-sectional survey in Germany. Sera collected from a representative cohort of adults (age 18–79; n = 6,663) in Germany were tested for anti-*T. gondii* IgG antibodies. Interview-derived data were used to evaluate associated factors. Multivariable logistic regression was applied using sampling weights and accounting for survey design cluster effects. Seroprevalence increased from 20% (95%-CI:17–23%) in the 18–29 age group to 77% (95%-CI:73–81%) in the 70–79 age group. Male gender, keeping cats and BMI ≥30 were independent risk factors for seropositivity, while being vegetarian and high socio-economic status were negatively associated. Based on these data, we estimate 1.1% of adults and 1.3% of women aged 18–49 to seroconvert each year. This implies 6,393 seroconversions annually during pregnancies. We conclude that *T. gondii* infection in Germany is highly prevalent and that eating habits (consuming raw meat) appear to be of high epidemiological relevance. High numbers of seroconversions during pregnancies pose substantial risks for unborn children. Efforts to raise awareness of toxoplasmosis in public health programs targeting to *T. gondii* transmission control are therefore strongly advocated.

Infection with the protozoan parasite *Toxoplasma gondii,* the causative agent of toxoplasmosis, is a very common human disease worldwide^1^,^2^. *T. gondii* persists lifelong in the affected host organism. In cats and other feline obligate hosts, parasites reproduce sexually and shed up to hundreds of millions of oocysts. They resist moderate environmental conditions, and contaminate water and soil where they undergo sporulation. The resulting form is responsible for infection via ingestion of either contaminated food, water or dust^3^,^4^. Additionally, parasites can be found as tissue cysts in all warm-blooded animals including livestock. Eating raw or undercooked infected meat is thus a second epidemiologically-relevant mode of transmission^5^. Transfer of parasites, e.g. through infected transplants or vertically in utero, is an additional route of transmission^1^,^2^.

In humans, most infections remain asymptomatic or manifest with mild flu-like symptoms; however, severe forms can occur^6^,^7^. These include congenital toxoplasmosis that can develop when a woman becomes primary infected with *T. gondii* during pregnancy^2^,^8^. Clinical surveys demonstrate that up to 20% of such maternal infections result in transplacental transmission, and that in 27% of the infected neonates specific symptoms develop^9^,^10^. Depending on the gestational age of the fetus at infection, predominantly retinochoroiditis, calcifications, hydrocephalus, psychomotoric and neurological disabilities, and fetal death can develop^6^,^7^.

After diagnosis of maternal infection, fast initiation of therapy can efficiently reduce the risk of transplacental transmission and thus lower the disease burden of the newborn^11^,^12^,^13^,^14^. However, timely diagnosis can only be achieved through systematic screening of pregnant women. This is currently recommended in France and Austria, but in the absence of strong evidence of its benefit, not in the UK and in Germany^15^.The reasons are diverse, but it is argued that the cost of the initial as well as follow up tests and the errors that arise are not outweighed by the small number of infections that can be prevented^16^ (see also Discussion).

*T. gondii* infection is also an important cause of visual impairment. Infection at ocular sites (retina and the choroid) causes lesions leading to retinal scarring^6^,^7^. In a population-based study in Britain, the lifetime risk of symptomatic *T. gondii*-associated ocular disease was determined to be 18 in 100,000 individuals^17^. In Germany, 4.2% of uveitis cases are thought to be due to *T. gondii* infections^6^.

Acute and reactivating infections in immunocompromised persons (e.g. AIDS patients or transplant recipients) can affect the central nervous system. An incidence of 3% of cerebral toxoplasmosis, associated with poor prognosis, was found among allogeneic hematopoietic stem cell transplant recipients^18^,^19^. Collectively, different manifestations of toxoplasmosis lead to a significant amount of years of life lost and many life years lived with sequelae, which constitute an exceptionally high disease burden of public health concern^20^,^21^,^22^,^23^.

Apart from the well-proven clinical entities, persistent infections with *T. gondii* are suspected to be connected to mood disorders^24^. In the United States National Health and Nutrition Survey, prevalence of serum anti-*T. gondii* IgG was not elevated in unipolar mood disorders, like depression, but was higher in a subset of respondents with a history of bipolar disorder^25^. However, the link between *T. gondii* infection and neurological changes in humans awaits experimental verification and thus remains highly controversial.

Congenital toxoplasmosis is mandatorily notifiable to the Robert Koch Institute, which is responsible for the implementation of data collection and processing of anonymized case data inside the infectious disease notification system of Germany. It is suspected that these data are subject to a high level of underreporting and under-ascertainment of disease. Thus, information on disease incidence is missing. Previous serosurveys for *T. gondii* are limited regarding representativeness and sample size. Available studies are based on convenience sampling (mainly pregnant females) and lack a random selection of participants.

Our objectives were thus to assess the seroprevalence of *T. gondii* IgG antibodies from the population-based German representative health interview and examination survey of adults (DEGS1)^26^, to identify and quantify the contribution of factors potentially associated with *T. gondii* seropositivity and to estimate the annual number of infections during pregnancy in Germany.

## Results

### Seroprevalence and associated factors

**Figure 1:**
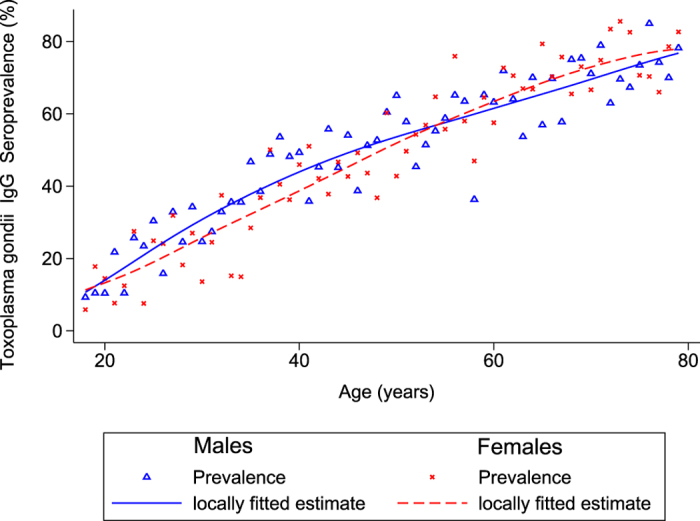
Estimated *T. gondii* seroprevalence, by age (18–79 years) and gender, in Germany, 2008–2011.

**Figure 2:**
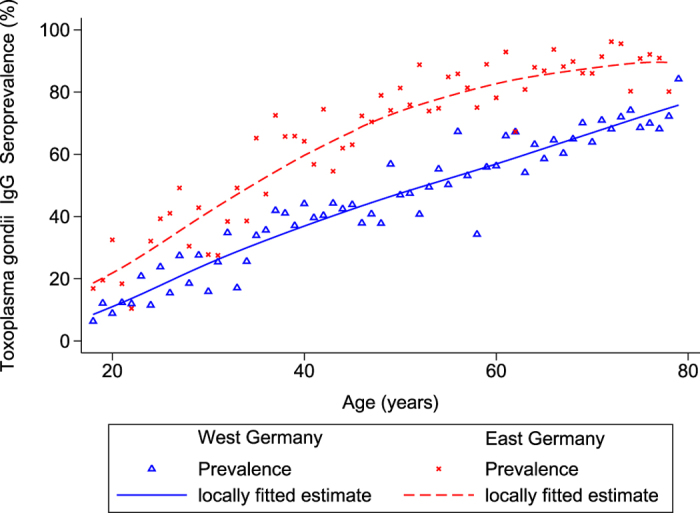
Estimated *T. gondii* seroprevalence, by geographical origin (East-West) and gender, in Germany, 2008–2011. Berlin is considered as East.

Altogether 6,663 participants with available blood samples were included in this study; 99 (1.5%) thereof had equivocal anti-*T. gondii* IgG titers of 4–7 and were thus excluded from the analysis. Out of 6,564 individuals, 3,602 (55%) were seropositive (Table 1). Seroprevalence increased from 20% (95%-confidence interval (95%-CI):17.1–23.1%) in the age group 18–29 to 77% (95%-CI:72.7–80.5%) in the 70–79 age group. Seroprevalence in participants older than 79 years (n = 116), not included in further analyses, was 84%. The observed seroprevalence increased at a rate of 1.09% with each year of age (Fig. 1). A significant interaction was noted between age and sex (p-value = 0.023), since higher seroprevalences were observed among younger males and older females. Male gender, although not associated in univariable analysis, was a significant risk factor in multivariable analysis (odds ratio (OR): 1.8; 95%-CI1.1–2.9). Age-related increase of seroprevalence was stronger amongst residents of East Germany when compared to the West (p-value of interaction term: <0.001) (Fig. 2). Prevalence in East Germany is more than 20% higher than in West Germany in the 40–69 age group.

Factors positively associated with seropositivity included cat contact. 15.8% of the participants indicated keeping a cat in the household, which proved to be a risk factor for seropositivity by multivariable analysis (OR: 1.3; 95%-CI:1.1–1.5), whereas holding a dog or other companion animal had no impact. Being overweight (25 ≤ BMI <30) or obese (BMI ≥30) was also positively associated with seropositivity (OR of 1.2; 95%-CI:1.0–1.5; and OR: 1.3; 95%-CI:1.0–1.6, respectively). People living in rural areas had an increased risk of infection (OR: 1.41; 95%-CI:1.1–1.8) compared to inhabitants of bigger cities. Factors negatively associated with *T. gondii* infection were being vegetarian (OR: 0.6; 95%-CI:0.4–1.0) and having a high socio-economic status (OR: 0.7; 95%-CI:0.6–0.9). No association could be found with diabetes.

### Incidence estimates of seroconversions

Using a regression model we estimated an annual incidence of 1,099 (95% CI:1,016–1,181) seroconversions per 100,000 adult inhabitants of Germany (Table 2). In women aged 18–49 (considered here as main age group potentially bearing children) this corresponds to 1,325 (95% CI: 1,007–1,642) seroconversions. Taking the age-dependent distribution of births among women (data from 2011) and an age-related increase in seroprevalence into account, it can be assumed that 74.1% of births were delivered by mothers susceptible to primary infection (“pregnancies at risk”). The susceptibility decreased from 90.8% in the age group 15–19 years to 52.6% in those 45–49 years old. The incidence of 1,325 in 100,000 women implied 6,393 predicted infections during the annual 662, 485 pregnancies in Germany (Table 3). These are 1.0% of all pregnant women and 1.3% of seronegative pregnant women (pregnancies at risk).

### Estimates of incidence of congenital toxoplasmosis

In a recent meta-analysis among patient cohorts, the rate of mother-to-child transmission among seroconverting pregnant women was reported to be 20% (95%-CI:15–26%)^9^. Extrapolated to the numbers derived here this would result in 1,279 (95% CI:959–1,662) annual cases of fetal infections in Germany. Based on estimates that 27% of infected neonates manifest with *T. gondii*-specific symptoms^10^, this would result in 345 neonates with clinical symptoms (congenital toxoplasmosis) annually in Germany.

### Depression

Participants in the survey who recalled an episode of depression during their lifetime had no association with seropositivity in univariable analysis (OR: 0.94; 95%-CI:0.78–1.13) (Table 4). This is confirmed by multivariable analysis adjusted for age and gender as well as for associated factors (OR: 0.84; 95%-CI:0.55–1.29) from analysis in Table 1.

## Discussion

This first nationwide representative seroprevalence study for *T. gondii* antibodies in Germany finds a high seroprevalence compared to other countries. Comparing our findings to previous data from Germany is hampered by the older studies’ lack of representativeness, but higher prevalences were found in a serosurvey among blood donors between 1994 and 1996 in North-East Germany (59%; n = 4,854; age 20–40)^27^, as well as in a study among pregnant women from South-West Germany (39%; n = 5,670; age 15–47)^28^. Thus our findings suggest a decrease in *T. gondii* infections in Germany over the last two decades. Similarly, in France, where comprehensive serosurveys reported a higher overall seropositivity in women of childbearing age (36.7%; n = 15,130; age 15–49), it decreased from 54.2% in 1995 to 43.8% in 2003 and to 36.7% in 2010^29^. In The Netherlands in 2006–2007, seroprevalence was significantly lower than in our study (26%; n = 5,541; age 0–79), decreasing from 41% in 1995/1996^30^. In the USA, seroprevalence declined from 14.1%; in 1988–1994 to 9.0% (n = 10,477; age 12–49) in 1999–2004^31^. These differences show that improvements for *T. gondii* infection prevention in Germany are necessary and also possible.

The case numbers from the German notification system are restricted to congenital toxoplasmosis and range between 23 cases in 2008 and 6 cases in 2014, without obvious time trends or regional clustering^32^. This is only a small fraction of cases we calculated in this study. Reasons for this discrepancy could be a high degree of under-ascertainment and under-reporting, as not all cases of congenital toxoplasmosis are laboratory confirmed, and laboratories do not have the clinical or demographic information to carry out the notification. As a result, current notification rates do not reflect the full picture of disease burden in Germany. Our extrapolations used manifestation rates from Li *et al.*^9^ and Dunn *et al.*^10^ for the calculation of incidence of congenital toxoplasmosis,which is independent of the influence of disease surveillance processes and therefore might provide a more accurate picture.

Calculations of the incidence of ocular and cerebral toxoplasmosis based on seroconversion rates are difficult, since information from surveys on manifestation rates is scarce, and the few sources available are outdated. Jones and Holland^33^ estimated, based on North American surveys in the 1970s, that 2% of *T. gondii*-infected individuals develop ocular toxoplasmosis. Extrapolated to incidence of seroconversion in Germany, this results in 22 patients of clinical ocular toxoplasmosis per 100,000 inhabitants annually. Due to an increase of immunosuppression in the aging population it can be assumed that incidence of cerebral toxoplasmosis is also increasing, although data on the population level are missing.

We found no evidence for a role for *T. gondii* in depression. This supports previous evidence from the population-based United States National Health and Nutrition Survey (NHANES III)^25^. Altogether, evidence on manifestation rates of all health outcomes of *T. gondii* infections is scarce and therefore studies have to be initiated to eliminate these limitations and to fully understand the disease burden of toxoplasmosis.

Seropositivity strongly increases in all age groups, indicating a strong force of infection also in the elderly. Frequent seroconversion in this group is particularly problematic since immunosuppression becomes more prominent with age. The observed increase is particularly strong in East Germany, arguing that the risk pattern changed after unification of Germany in 1990. Alternatively, this could be explained by a birth cohort effect of a previous time frame, when a higher risk of seroconversion resulted in higher seroprevalence today in older age groups. Although not associated in univariable analysis, men have a 1.76-times higher chance of being seropositive in multivariable analysis. This can be explained by effect modification of the factors higher socio-economic status, cat holding and male gender included in the final model. Since male Germans eat about twice as much meat and meat products as females^34^, the higher seroprevalence we observe in men can be reconciled with these eating habits. In our study, seroprevalences for *T. gondii* vary between regions in Germany. The consumption of freshly prepared raw minced meat (beef and pork), known in Germany as “Hackepeter” or “Mett”, is more widespread in East compared to West Germany^35^,^36^. This may explain the higher seroprevalence in East Germany and additionally confirms that raw meat poses a substantial risk for infection with *T. gondii*^4^,^37^.

Furthermore, our results show that overweight and obese study participants have an increased chance of becoming seropositive, consistent with recent data from a smaller study in Germany^38^. There is good evidence that higher meat consumption is connected to an increase in body weight^39^,^40^,which in turn leads to a higher chance to ingest contaminated meat. Eating vegetarian, on the other hand, is negatively associated with seropositivity, arguing that consumption of oocyst-contaminated vegetables is not a major driver of seroconversion. Altogether, these data are consistent with the majority of infections being food-borne^4^,^37^,^41^.

Some previous epidemiological studies did not observe an elevated risk for cat owners^37^,^42^, while representative serosurveys in The Netherlands have reported such an association^30^,^43^. A case-control study in the USA reported a higher risk for cat owners with 3 or more cats^44^. Cats are biologically essential in the life cycle of *T. gondii,* but cat exposure seems to be a less important factor compared to exposure to contaminated food, in particular meat. Nevertheless, prevention of infection through contact with cats is possible since they shed oocysts only for 1–3 periods during lifetime, and sporulation can be avoided by daily removal of cat litter^45^.

The strengths of our study include a large sample size with a sampling process leading to a representative study population, with an appropriate adjustment for demographic variables. As the DEGS1 study recruited only the adult population, we have no data on infants and children. This deficiency should be addressed in the future. The analyses in our study are based on seroprevalence data, but seroconversion is not equivalent to clinical manifestation of disease. The associated factors in our study are mostly contextual factors. Specific and proximal factors like lifetime cat exposures or lifetime raw meat consumption were not available from the survey. The same is true for the association to lifetime depression, which is only one mood disorder hypothesized to be connected to *T. gondii* infections.

Toxoplasmosis is hard to combat from the public health point of view and often neglected in programs targeting food-borne diseases^46^. Primary prevention of infections is important and can prevent a significant proportion of disease cases of all clinical manifestations^47^. Since a *T. gondii* infection can reactivate upon immune suppression, it should be avoided in all population groups to prevent subsequent ocular or cerebral disease later in life.

Secondary prevention in the form of a repeated serological screening during pregnancy might be successful to prevent further pregnancy-associated cases. Arguments for serological screening have to consider the positive predictive value of the screening tests and the risk-benefit ratio of medication used to treat infection. The effectiveness of prenatal screening and treatment is debated, largely because of different opinions on cost-benefit^48^,^49^, health consequences of the screening for the mother^50^, and the effectiveness of the treatment^16^,^51^. Nevertheless, early serological screening of pregnant women to detect maternal seroconversion, followed by rapid treatment in utero (as is done in France and Austria) may prevent transplacental transmission and thus neonatal infection and clinical manifestation^11^,^12^,^13^,^14^. In these countries, the key for success seems to be frequent re-testing using highly sensitive PCR analysis of appropriate maternal samples and rapid treatment^2^,^52^. This practice might explain the striking differences in percentages of observed hydrocephalus cases in newborns of congenital infections seen between France and the US (0.3–0.8% vs. 31%)^53^,^54^,^55^. In the latter country, this regimen is not practiced. In Austria, with its systematic prenatal screening program, a 6-fold decrease in transmission compared to untreated women was recently reported^56^. At the rates of congenital infection reported here, screening is considered to be cost-saving^57^. In Germany, screening tests to detect *T. gondii* infection during pregnancy are currently not covered by statutory health insurance, after scientific evaluation of its pros and cons by their medical services^58^. As a result, pregnant women have to self-pay for these tests. This significantly affects the testing rate, especially in women of lower socioeconomic status^59^.

Patient numbers and preventable cases are expected to be significantly higher than can be inferred from the notification system. It should be re-evaluated whether serological testing of pregnant women should be offered by physicians and paid for by health insurance in Germany. Moreover, women of childbearing age and those immunocompromised should be more specifically targeted and informed about potential risks, with emphasis on the prevention of food-borne infection pathways. Finally, veterinary services, the meat industry and agriculture should continue their efforts to reduce *T. gondii* in meat, especially pork, as this is frequently eaten raw in Germany.

## Methods

The German health interview and examination survey of adults (DEGS1) was a representative, nationwide cross-sectional survey conducted between 2008 and 2011. Detailed study descriptions have been presented previously^26^. Briefly, it followed a stratified two-stage cluster sampling strategy to assess the health status of adults between 18 and 79 years of age in Germany. Three professionally trained teams each visited a total of 180 sample points. The sample points are distributed across Germany according to federal state and municipality size in order to reflect the distribution of the German population. After written informed consent 7,988 persons (age 18–79) participated in DEGS1. These were interviewed with a standardized questionnaire and blood samples were given. The response rate among those approached was 48.4% and the analysis of non-responder questionnaires revealed high population representativeness among participants. Interview data were used to assess potential risk factors for positive outcome^26^. DEGS1 was approved by the ethical review board of the Charité Medical School, Berlin, Germany (No. EA2/047/08) and all methods were carried out in accordance with the approved guidelines.

Aliquots of sera (thawed twice) from DEGS1 were analyzed for the presence of anti-*T. gondii* IgG antibodies by a commercial automatic and quantitative enzyme-linked fluorescence assay (ELFA; VIDAS TOXO IgG II; Biomérieux SA, France) of the same batch according to the manufacturer’s instructions. In comparative studies, sensitivity of the assay was shown to be above 99% and specificity above 98%^60^. Titers of 0–3 were considered negative, 4–7 equivocal, and ≥8 positive. We excluded all samples with equivocal test results (1.5%) from the analysis.

Univariable and multivariable logistic regression used sampling weights, accounted for the cluster structure of the survey design and checked for effect modifications. Survey weights based on age, sex, residence in West or East Germany and nationality (German vs. non-German) were calculated to correct for deviations from the German population statistics (December 31, 2010) and used throughout the analysis.

We investigated associations between explanatory variables and seropositivity by univariable logistic regression analyses. The most frequent category was selected as the reference category except for variables where a norm category was available. All variables below p-value of 0.15 were included in a multivariable logistic regression model. As seropositivity regarding *T. gondii* is cumulative and therefore strongly related to age, all two-way interactions involving age were included. Age-related prevalence was smoothed by using the Lowess procedure of the program Stata 12.1 and graphed stratified for sex and East-West geographical origin. The annual incidence of seroconversions was calculated from linear regression using the one-year age group as independent and weighted prevalence as dependent variable. The annual number of seroconversions among pregnant women between 15 and 49 years of age in Germany was calculated by using the number of births in each one-year age group of mothers multiplied by the expected proportion of susceptibles, considering an annual incidence of 1,325 in 100,000 women, and corrected for the mean duration of pregnancies (266 days). For the age group 15 to 17 years, prevalence estimates were derived from the study data, assuming a constant increase as in the age group 18 to 25 years.

For the calculation of seroconversions among pregnancies, data on number of women and number of childbirths by age of the mother for 2011 were retrieved from the German Federal statistical office (https://www.destatis.de/). In the calculations we regarded multiple births by looking at how many children each respondent had.

## Additional Information

**How to cite this article**: Wilking, H. *et al.* Prevalence, incidence estimations, and risk factors of *Toxoplasma gondii* infection in Germany: a representative, cross-sectional, serological study. *Sci. Rep.* **6**, 22551; doi: 10.1038/srep22551 (2016).

## Figures and Tables

**Table 1:** Stratified seroprevalence of IgG antibodies against *T. gondii* detected by ELFA in adults aged 18 to 79 years and results of weighted logistic regression analysis of potential risk factors for seropositivity, 2008–2011.

Characteristic (total no.)^a^	n (pos)^a^	Prevalence in % (95% CI)	Univariable analysis		Multivariable analysis	
			Odds ratio (95% CI)	p-Value	Odds ratio (95% CI)	p-Value
Sex						
Women (n = 3,443)	1,864	48.85 (46.56–51.14)	ref	ref	ref	ref
Men (n = 3,121)	1,738	49.31 (46.35–52.27)	1.02 (0.90–1.15)	0.767	1.76 (1.08–2.91)	0.023
Interaction term sex*age	–	–	–	–	0.99 (0.98–1.00)	0.024
Age						
Yearly	–	–	1.05 (1.05–1.06)	<0.001	1.06 (1.05–1.06)	<0.001
Age group (years)						
18–29 (n = 994)	214	19.95 (17.10–23.13)	ref	ref	–	–
30–39 (n = 790)	301	35.46 (31.01–40.17)	2.21 (1.70–2.85)	<0.001	–	–
40–49 (n = 1,212)	628	48.10 (44.57–51.64)	3.72 (3.00–4.60)	<0.001	–	–
50–59 (n = 1,298)	796	57.97 (54.25–61.61)	5.54 (4.37–7.01)	<0.001	–	–
60–69 (n = 1,267)	903	69.48 (65.13–73.50)	9.13 (7.01–11.89)	<0.001	–	–
70–79 (n = 1,003)	760	76.82 (72.74–80.45)	13.30 (10.20–17.34)	<0.001	–	–
Residence (East-West)^b^						
West (n = 4,484)	2,067	44.02 (42.10–45.96)	ref	ref	ref	ref
East (n = 2,080)	1,535	68.17 (64.13–71.95)	2.72 (2.24–3.30)	<0.001	0.98 (0.56–1.71)	0.936
Interact term East*age	–	–	–	–	1.03 (1.02–1.04)	<0.001
Residence (north-south)^c^						
North (n = 1,683)	1,029	53.90 49.94–57.81)	1.46 (1.20–1.79)	<0.001	1.17 (0.93–1.46)	0.177
Middle (n = 2,879)	1,650	50.04 (46.12–53.96)	1.26 (1.02–1.53)	0.026	0.98 (0.80–1.20)	0.859
South (n = 2,002)	923	44.38 (41.23–47.58)	ref	ref	ref	ref
Population of municipality						
<5,000 (n = 1,171)	725	56.21 (49.89–62.35)	1.67 (1.27–2.19)	<0.001	1.41 (1.11–1.79)	0.005
5,000–<20,000 (n = 1,598)	904	52.11 (47.50–56.69)	1.41 (1.14–1.76)	0.002	1.18 (0.97–1.44)	0.100
20,000–<100,000 (n = 1,941)	975	43.50 (40.35–46.71)	ref	ref	ref	ref
>100,000 (n = 1,854)	998	48.72 (44.68–52.79)	1.23 (1.01–1.51)	0.043	1.17 (0.96–1.43)	0.118
Pet in household^d^						
No pet (n = 4,360)	2,400	49.00 (46.64–51.36)	ref	ref	–	–
Any pet (n = 2,045)	1,099	48.46 (45.38–51.55)	0.98 (0.87–1.10)	0.718	–	–
Dog^d^						
No (n = 5,588)	3,070	49.19 (46.93–51.46)	ref	ref	–	–
Yes (n = 806)	424	46.66 (41.94–51.45)	0.90 (0.75–1.09)	0.288	–	–
Cat^d^						
No (n = 5,383)	2,936	48.37 (45.99–50.76)	ref	ref	ref	ref
Yes (n = 1,011)	558	51.46 (47.70–55.21)	1.13 (0.97–1.32)	0.127	1.27 (1.06–1.51)	0.009
Other animals^d^						
No (n = 5,679)	3,126	49.29 (47.04–51.54)	ref	ref	–	–
Yes (n = 715)	368	45.73 (40.62–50.93)	0.87 (0.71–1.06)	0.168	–	–
Eating vegetarian^d^						
No (n = 6,148)	3,413	49.74 (47.54–51.93)	ref	ref	ref	ref
Yes (n = 248)	94	35.77 (28.21–44.11)	0.56 (0.40–0.80	0.002	0.62 (0.42–0.99)	0.048
Body mass index (BMI)						
Underweight (BMI < 18.5) (n = 86)	22	23.95 (14.44–37.02)	0.50 (0.27–0.94)	0.031	0.66 (0.34–1.28)	0.212
Normal weight (18.5 ≤ BMI < 25) (n = 2,430)	1,063	38.44 (35.83–41.12)	ref	ref	ref	ref
Overweight (25 ≤ BMI < 30) (n = 2,457)	1,470	54.94 (52.08–57.76)	1.95 (1.70–2.24)	<0.001	1.23 (1.03–1.47)	0.024
Obesity (BMI ≥ 30) (n = 1,552)	1,023	58.99 (55.53–62.39)	2.35 (2.15–2.55)	<0.001	1.28 (1.01–1.55)	0.048
Diabetes (12 month prevalence)						
No (n = 6,072)	3,253	47.92 (45.76–50.10)	ref	ref	ref	ref
Yes (n = 432)	313	67.12 (60.87–72.82)	2.22 (1.70–2.90)	<0.001	0.82 (0.61–1.10)	0.181
Socio-economic status^e^						
Low (n = 1,034)	617	54.93 (50.97–58.83)	1.23 (1.03–1.47)	0.024	1.20 (0.95–1.52)	0.132
Middle (n = 3,933)	2,217	49.77 (47.13–52.43)	ref	ref	ref	ref
High (n = 1,553)	745	41.42 (38.09–44.83)	0.71 (0.61–0.83)	<0.001	0.72 (0.60–0.85)	<0.001
Total (n = 6,564)	3,602	49.08 (46.92–51.23)	–	–	–	–

^a^unweighted.

^b^Eastern states: Berlin, Brandenburg, Mecklenburg-West Pomerania, Saxony, Saxony-Anhalt, Thuringia. Western states: Baden-Württemberg, Bavaria, Bremen, Hamburg, Hesse, Lower Saxony, Northrhine-Westfalia, Rhineland-Palatinate, Saarland, Schleswig-Holstein.

^c^Northern states: Schleswig-Holstein, Hamburg, Lower Saxony, Bremen, Berlin, Brandenburg, Mecklenburg-West Pomerania. Middle states: Nordrhine-Westfalia, Hesse, Saxony, Saxony-Anhalt, Thuringia. Southern states: Rhineland-Palatinate, Baden-Württemberg, Bavaria, Saarland.

^d^at day of interview.

^e^Socio-economic status is measured using a score composed of income, education and professional status.

**Table 2:** Annual incidence of seroconversions of IgG antibodies against *T. gondii* stratified by sex and age groups.

Age group	Annual incidence in 100,000 persons	95% confidence interval
Women		
18–79 years	1,189	1,077–1,301
18–49 years	1,325	1,007–1,642
50–79 years	944	620–1,268
Men		
18–79 years	1,007	897–1,117
18–49 years	1,436	1,186–1,686
50–79 years	794	454–1,134
Total	1,099	1,016–1,181

**Table 3:** Seroprevalence of *T. gondii* infection by age group in women of childbearing age.

Age of the mother at child birth^a^	Number of women in German population^b^	Annual expected number of seroconversions^c^	Number of births in Germany^b^	Proportion of women giving birth (in %)	Proportion of seronegative women (in %)^d^	Annual expected number of seroconversions among pregnancies (% of pregnancies at risk)
15–19	1,985,672	26,310	16,459	0.83	90.8	159 (1.1)
20–24	2,421,962	32,091	88,777	3.67	84.5	857 (1.1)
25–29	2,447,109	32,424	191,010	7.81	78.1	1,843 (1.2)
30–34	2,437,824	32,301	222,218	9.12	71.7	2,144 (1.3)
35–39	2,359,922	31,269	115,634	4.90	65.4	1,116 (1.5)
40–44	3,116,101	41,288	27,131	0.87	59.0	262 (1.6)
45–49	3,490,840	46,254	1,256	0.04	52.6	12 (1.8)
Total	18,259,430	241,937	662,485	–	74.1	6,393 (1.3)

^a^Estimates for the 15–17 year-old were derived from the study data assuming constant increase as in the age group 18 to 25 years.

^b^German Federal statistical office, 2011.

^c^at an incidence of 1,325 in 100,000 inhabitants.

^d^inverse of prevalence, estimates from the regression model.

**Table 4:** Stratified seroprevalence of IgG antibodies against *T. gondii* detected by ELFA in adults aged 18 to 79 years, and results of weighted logistic regression analysis of seropositivity on depression as an outcome, 2008–2011.

Lifetime depression (total no.)^a^	n (pos)^a^	Prevalence (95% CI)	Univariable analysis		Multivariable analysis^b^		Multivariable analysis^c^	
			Odds ratio (95% CI)	p-Value	Odds ratio (95% CI)	Odds ratio (95% CI)	Odds ratio (95% CI)	Odds ratio (95% CI)
No (n = 5,747)	3,161	49.12 (46.90–51.34)	ref	ref	ref	ref	ref	ref
Yes (n = 768)	409	47.56 (43.05–52.11)	0.94 (0.78–1.13)	0.497	1.05 (0.84–1.19)	0.365	0.84 (0.55–1.29)	0.420

^a^unweighted, lifetime incidence of depression (self-reported) was assessed by asking the closed question “Have you ever been diagnosed with depression by a physician or a psychotherapist”?

^b^adjusted for age, sex and interaction between both.

^c^adjusted for variables in multivariable model of Table 1.
